# The Role of MATN3 in Cancer Prognosis and Immune Infiltration Across Multiple Tumor Types

**DOI:** 10.7150/jca.103523

**Published:** 2025-01-27

**Authors:** Chongjiu Qin, Haifei Qin, Haixiang Xie, Yuhua Li, Aoyang Bi, Xiwen Liao, Kejian Yang, Chunmiao Lu, Tao Peng, Guangzhi Zhu

**Affiliations:** 1 Department of Hepatobiliary Surgery, The First Affiliated Hospital of Guangxi Medical University, Nanning, 530021, Guangxi Zhuang Autonomous Region, People's Republic of China.; 2 Guangxi Key Laboratory of Enhanced Recovery after Surgery for Gastrointestinal Cancer, 530021, Nanning, People's Republic of China.; 3 Key Laboratory of early Prevention & Treatment for regional High Frequency Tumor (Guangxi Medical University), Ministry of Education, Nanning, 530021, Guangxi Zhuang Autonomous Region, People's Republic of China.

**Keywords:** MATN3, pan-cancer, immunization, diagnosis, prognosis

## Abstract

**Background:** MATN3 is a member of the matrix protein family and is involved in the regulation of osteoarthritis as well as the development of gastric cancer. We investigated the role of MATN3 in pan-cancer and validated this result by *in vitro* experiments.

**Material and Methods:** We applied multiple databases to explore the expression of MATN3 in 33 types of tumors. Kaplan-Meier survival analysis is performed to understand the effect of MATN3 on Prognostic value in patients with different cancer types. The TIMER database was applied to explore the relationship between MATN3 and immune checkpoint genes, immunomodulatory genes, and immune infiltration, the Sanger box was applied to explore the relationship between MATN3 and methylation, the Genomic Cancer Analysis database was utilized to explore the relationship between MATN3 expression and pharmacological sensitivity, and the STRING database was used to explore the co-expressed genes and to complete the Gene Ontology and Kyoto Encyclopedia of Genes and Genomes pathway enrichment analysis. Data from The Cancer Genome Atlas as well as Genotype-Tissue Expression databases were statistically analyzed and visualized using the R software. Immunohistochemistry and Western blotting for detection of MATN3 expression. CCK-8 and clone formation were used to detect cell proliferation, Wound-healing assay and transwell invasion were used to detect cell migration and invasion ability.

**Results:** MATN3 is overexpressed in most cancer types, indicating a poorer prognosis. It is closely linked to methylation, immunomodulatory genes, and immune checkpoint genes, contributing to immune infiltration in various cancer types. *In vitro* experiments showed that silencing MATN3 inhibited cell proliferation, migration, and invasion ability.

**Conclusions:** MATN3 is involved in the immune infiltration of cancer and affects the prognosis of many cancer types, and can be used as an immune as well as prognostic biomarker for pan-cancer.

## Introduction

According to the World Health Organization's (WHO) annual report in 2022, there were an estimated 20 million new cancer cases and 9.7 million cancer deaths globally, making cancer the leading cause of human death^1^. Current tumor treatment primarily relies on surgical resection combined with radiotherapy, chemotherapy, immunotherapy, and targeted therapy, which has significantly improved patient survival rates and reduced recurrence rates^2^. However, the toxic side effects of drugs and the emergence of drug resistance pose significant challenges, highlighting the urgent need for new cancer diagnostic biomarkers to aid in early detection, diagnosis, and treatment. MATN3 belong to the extracellular matrix (ECM) protein family and encode components of the vascular hemophilia A structural domain^3^. Previous studies have implicated MATN3 in the development and progression of osteoarthritis and its potential role in gastric cancer invasion^4^. Nevertheless, previous studies have carried out preliminary exploration of gastric cancer, but there is still a lack of understanding at the pan-cancer level. We examined its association with cancer diagnosis, prognosis, molecular typing, immune subtyping, methylation, drug sensitivity, and immune infiltration, providing insights into the multifaceted role of MATN3 in cancer. In addition, we also knocked down MATN3 in liver cancer cell lines Huh7 and Hep3B to study the effect of MATN3 on the function of liver cancer cells.

## Materials and Methods

### Gene expression analysis of MATN3

TIMER 2.0 (http://timer.cistrome.org) is a free, open-access data sharing platform where users can explore immune infiltration in cancer types and examine the expression of target genes in tumor and normal tissues across various cancer types^5^. We acquired RNA-seq data for 33 types of cancer from The Cancer Genome Atlas (TCGA) database (http://cancergenome.nih.gov) and the Genotype-Tissue Expression (GTEx) database (http://commonfund.nih.gov/GTEx/). We utilized data from both databases to investigate the differential expression of MATN3 across various cancer types. The relationship between MATN3 expression and clinicopathological features of HCC in the TCGA database is shown in the table (Table S2). Box plots were employed to visualize the distribution of gene expression levels. Furthermore, we investigated the association between MATN3 expression and clinicopathological features (including T-stage, N-stage, M-stage, and histologic grading), with statistical significance evaluated using the Wilcoxon test. Additionally, we utilized the pROC software package in R language (version 4.2.1) to construct receiver operating characteristic (ROC) curves to assess the diagnostic value of MATN3 across 33 cancer types^6^. Statistical significance is indicated as (*: p-value < 0.05; **: p-value < 0.01; ***: p-value < 0.001), and normal data are shown in gray columns when available.

### Prognostic analysis of pan-cancer in MATN3

We obtained information on the clinical characteristics of 33 cancer types in TCGA and evaluated the correlation between differential expression of MATN3 and prognosis. The data were categorized into MATN3 high-expression and MATN3 low-expression groups based on the median MATN3 expression value, one-way COX analysis and Kaplan-Meier curves were used to evaluate the significance of differential expression of MATN3 for the three aspects of survival (OS), disease-specific survival (DSS), and progression-free interval (PFI) for the 33 cancer types, and a forest plot was used to demonstrate the results of the one-way COX analysis^7^.

### Molecular and immunological subtyping

We explored the relationship between MATN3 expression and the molecular and immune subtypes of different tumors at TISIDB (http://cis.hku.hk/TISIDB/)^8^.

### Correlation of MATN3 expression with immune checkpoint genes, immunomodulatory genes, and drug sensitivity

Tumor treatment is closely related to the expression of immune checkpoint genes and immunomodulatory genes, and we applied the TIMER database to explore the correlation between MATN3 expression and immune checkpoint genes and immunomodulatory genes^9^. The GSCA database (https://guolab.wchscu.cn/GSCA/#/) was used to explore the MATN3 expression and drug sensitivity Relationship^10^.

### Association of MATN3 with immune infiltration and gene mutations

The TIMER database was used to explore the relationship between MATN3 expression and immune infiltrating cells in 33 cancer types. We explored the relationship between six types of cells (B cell, T cell CD4, T cell CD8, Neutrophil, Macrophage, and DC) and MATN3 expression, and the results are presented in the form of heatmaps, and Pearson's correlation coefficient was used to describe the correlation^9^. Additionally we explored the relationship between MATN3 and tumor purity, the ESTIMATE algorithm was used to calculate stromal scores, immune scores, and estimation scores for the relevant tumor samples and to assess the correlation between the level of MATN3 expression and these scores. Additionally pearson correlation was used to explore the correlation of MATN3 expression with for microsatellite instability (MSI)^11^ as well as tumor mutational load (TMB)^12^. We also discussed MATN3 gene alterations in pan-cancer using the cBioPortal database^13^.

### Relationship between MATN3 expression and methylation

Promoter methylation is closely related to cancer development, and the level of promoter methylation is closely related to the clinical features and prognosis of tumors^14^. In addition to this, cancer development is also associated with m6A, m5C and m1A modifier genes^15^. We explored the expression of MATN3 promoter methylation levels in different cancer types by UALCAN (https://ualcan.path.uab.edu/cgi-bin/ualcan-res.pl)^16^. SAMRT (http://www.bioinfo-zs.com/smartapp/) was used to probe the distribution of chromosomal methylation probes^17^. We also explored the correlation between MATN3 mRNA expression and genes related to methylation modifications. MethSurv (https://Biit.cs.ut.ee/MethSurv/) was used to probe DNA methylation survival prognosis^18^.

### Enrichment analysis

We utilize the STRING database (https://cn.string-db.org/)^19^. We constructed a PPI network of MATN3 with 57 related genes PPI interaction network of MATN3 with 57 related genes was constructed, in addition, we also constructed GGI interaction network of MATN3 with 21 related genes from GeneMANIA database (http://genemania.org/)^20^, and GO and KEGG enrichment analyses were performed based on the co-expressed genes of MATN3.

### Cell lines and cell culture

Hep3B and Huh7 were purchased from the National Center for the Preservation of Certified Cell Cultures (https://www.cellbank.org.cn/), and Hep3B and Huh7 were cultured in MEM containing 10% FBS (Gibco, Shanghai, China).

### Cell transfection

Small/short interfering RNAs (siRNAs) designed by Sangon Biotech (sangon.com) to interfere with MATN3, the sequence is shown in the table (Table S3). The siRNA was transfected into cells using the bio-engineering transfection reagent transmate, and the transfection efficiency was verified by WB experiments.

### Western blot analysis

Proteins were extracted according to the instructions of the reagent supplier, protein concentration was measured by BCA method, proteins were bound to PVDF membrane after electrophoresis and membrane transfer operation, incubated with primary antibody at 4℃ overnight, and then incubated with corresponding goat anti-rabbit antibody. Primary antibody concentration: MATN3 (1:2000, Baijia, China).

### Cell proliferation and colony formation assay

The corresponding hepatocellular carcinoma cells were planted into 96-well plates at a density of 3000 cells/well and tried with CCK-8 kit according to the instructions of the reagent supplier (HC0854, WILBER) In order to evaluate the effect of MATN3 on colony formation, we planted the cells into 6-well plates at a density of 6,000 cells per well, and the experiments were repeated three times.

### Immunohistochemical analysis of MATN3 in hepatocellular carcinoma

To assess the disparity in MATN3 expression between tumor tissues and normal tissues, we recruited 30 hepatocellular carcinoma patients who underwent surgery at the Department of Hepatobiliary Surgery of the First Affiliated Hospital of Guangxi Medical University. Here are their detailed clinical parameters (Table S1). These patients were diagnosed with hepatocellular carcinoma based on postoperative pathology. We obtained informed consent from each patient, who then signed an informed consent form prior to collecting surgical specimens^21^ with the authorization of the Ethical Review Committee of the First Affiliated Hospital of Guangxi Medical University (authorization code: 2024-E295-01). Inclusion criteria: 1. Patients with primary hepatocellular carcinoma treated for the first time; 2. Patients treated with partial hepatectomy; 3. Patients who have not undergone interventional therapy, targeted therapy and immunotherapy before surgery. Exclusion criteria: Patients with a history of other tumors besides hepatocellular carcinoma. The experimental process was carried out according to the instructions of the reagent supplier, and the specific operation details were described above^22^. The antibody used in the study was MATN3 (Solarbio, dilution concentration: 1:200). Five randomly selected fields of view were observed and semi-quantitatively scored for MATN3, with the score equal to the intensity of expression multiplied by the area of expression. Expression intensity scores ranging from 0-3 indicate negative, weak staining (light yellow), moderate staining (light brown), and strong staining (dark brown), respectively. Expression area scores ranging from 0-4 represent <5%, 6-25%, 26-50%, and 51-75% >75%, respectively. The degree of positive staining was defined as: 1-3 as weakly positive (+); 4-6 as moderately positive (+++); and 7-12 as strongly positive (++++)^23^.

### Wound healing assay

Cells were inoculated in 6-well plates and scratched with a 20ul lance tip when single cell confluence reached 90%. Photographs were taken at 0H, 24H and 48H for observation respectively. The experiment was repeated three times.

### Cell migration assay

Experiments were performed using transwell chambers with 8 µm pore size. Serum-free DMEM medium containing 8×10^4^ cells was inoculated into the upper chamber, and the lower chamber was injected with DMEM containing 10% FBS for 24 h. After 24 h, paraformaldehyde fixation was performed, crystal violet staining was performed and the number of cells passing through the bottom of the chambers was recorded when placed under a microscope.

## Results

### Expression of MATN3

**Figure 1:**
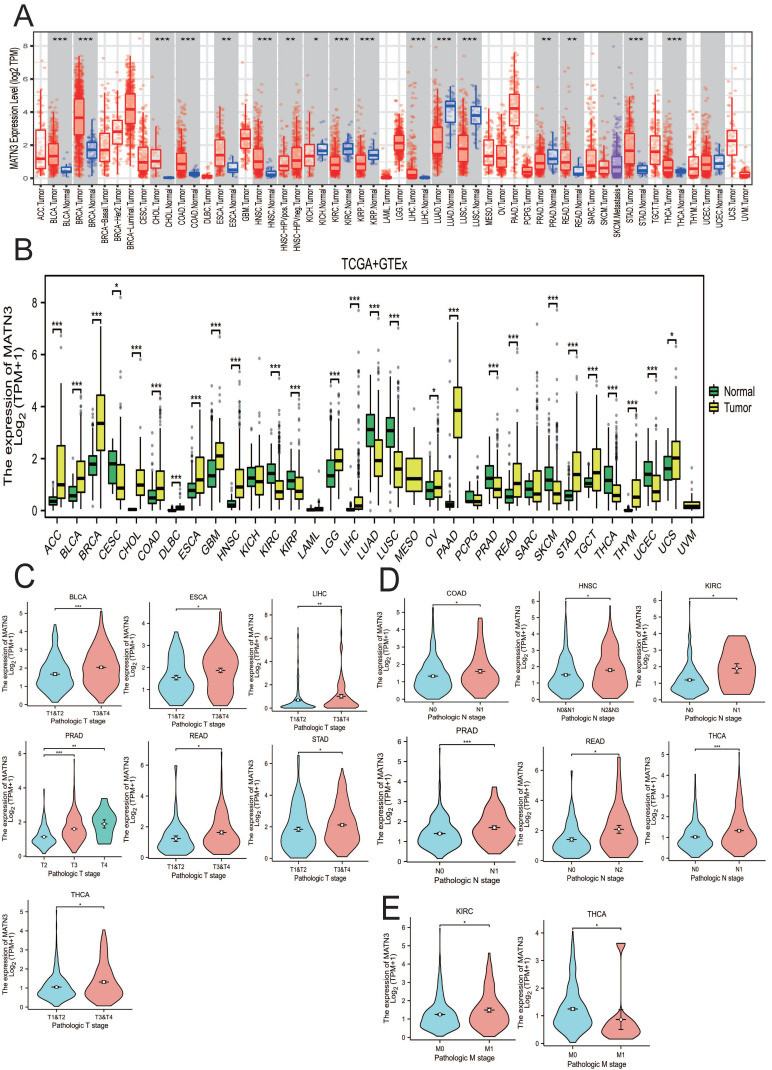
MATN3 expression in pan-cancer from different databases. (**A**) MATN3 expression in pan-cancer in TIMER2.0. (**B**) MATN3 expression in cancer types in TCGA + GTEx. (**C**) The relationship of MATN3 expression to T stage in BLCA, ESCA, LIHC, PRAD, READ, STAD and THCA. (**D**) The relationship of MATN3 expression to N stage in COAD, HNSC, KIRC, PRAD, READ, THCA. (**E**) The relationship of MATN3 expression to M stage in KIRC, THCA.

We obtained the expression of MATN3 in tumor tissues and normal tissues from TIMER2.0 in a variety of cancer types (Figure 1A), and combined the TCGA database + GTEx database to explore the expression of MATN3 in pan-cancer (Figure 1B). In addition, we also explored the expression of MATN3 in different clinicopathologic features (including T-stage, N-stage, M-stage, and histologic grading) (Figure 1C, Figure 1D). From (Figure 1A), it can be seen that bladder uroepithelial carcinoma (BLCA), breast invasive carcinoma (BRCA), cholangiocarcinoma (CHOL), colon adenocarcinoma (COAD), esophageal carcinoma (ESCA), head and neck squamous cell carcinoma (HNSC), hepatocellular carcinoma (LIHC), rectal carcinoma (READ), gastric carcinoma (STAD), thyroid carcinoma (THCA), and endometrioid carcinoma (UCEC) were significantly up-regulated compared to normal tissue expression next to the tumor, but DBF4B expression was down-regulated in renal smoky cell carcinoma (KICH), renal clear cell carcinoma (KIRC), renal papillary cell carcinoma (KIRP), lung adenocarcinoma (LUAD), lung squamous cell carcinoma (LUSC), and prostate adenocarcinoma (PRAD) (Figure 1B). Subsequently, we also explored the relationship between MATN3 expression and pathologic features; for T stage (Figure 1C), we found that MATN3 expression was expressed at high levels in the higher T stage of BLCA, ESCA, LIHC, PRAD, READ, STAD, and THCA as compared to the lower T stage. For N stage (Figure 1D), MATN3 expression was higher in the higher N stage of COAD, HNSC, KIRC, PRAD, READ, and THCA. For M stage (Figure 1E), MATN3 expression was higher in the higher staging of KIRC, but the opposite was true in THCA. We also found that MATN3 had high diagnostic value in all 15 cancer types (Figure S1) and could play a great role in cancer diagnosis. The names of the 33 cancer types along with their abbreviations are shown in the Abbreviations section. Univariate logistic regression analysis (Table S2) showed a strong correlation between MATN3 expression and some clinicopathological features in HCC, especially Age (OR=0.602, 95%CI = 0.400-0.907). p=0.015), Pathologic stage (OR=1.833, 95%CI =1.126-2.985, p=0.015) and Pathologic T stage (OR=1.723, 95%CI =1.069-2.777, p=0.025).

### Prognostic analysis of pan-cancer in MATN3

**Figure 2:**
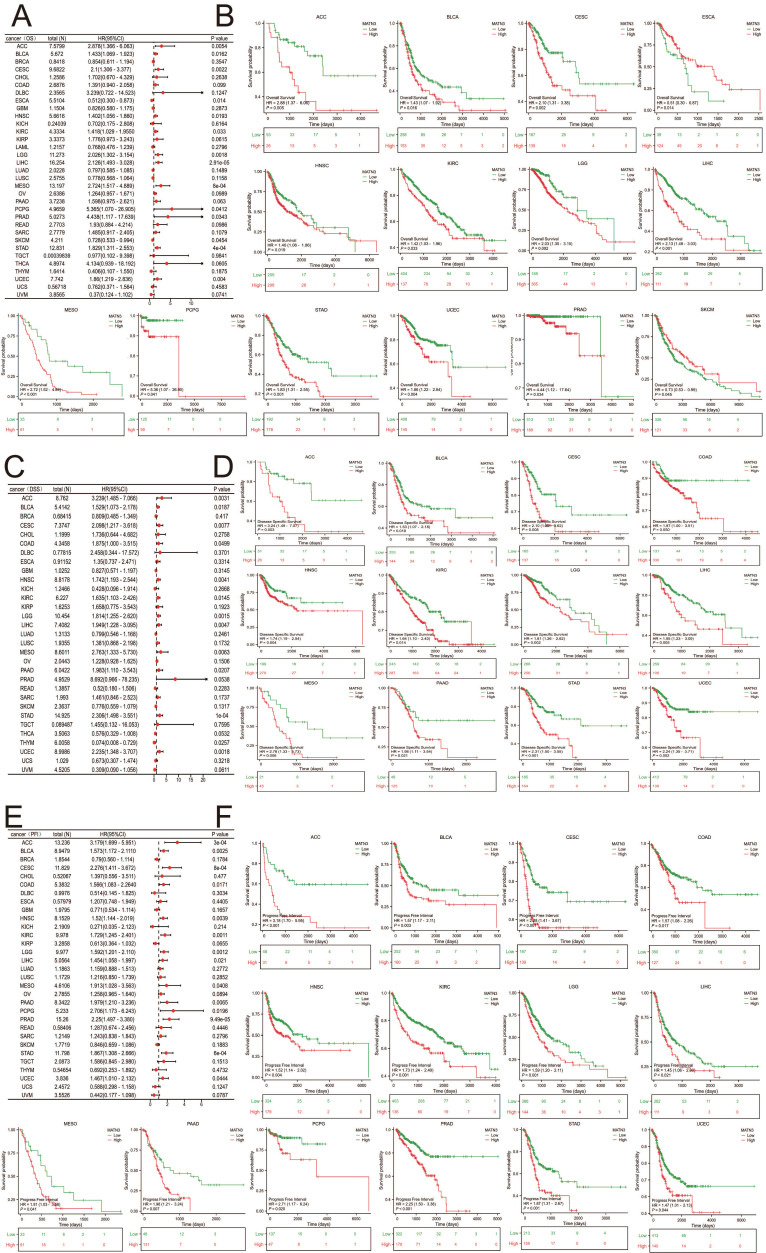
The univariate regression and Kaplan-Meier curves for OS (A, B), DSS (C, D), and PFI (E, F) in pan-cancer.

Three indicators, OS, DSS, and PFI, were used to evaluate MATN3 and cancer prognosis. The relationship between the differences in MATN3 expression and OS, DSS, and PFI was demonstrated in forest plots (Figure 2A, Figure 2C, and Figure 2E). For OS, high expression of MATN3 was associated with poor prognosis in ACC, BLCA, CESC, HNSC, KIRC, LGG, LIHC, MESO, PCPG, PRAD, STAD, UCEC. However, opposite results were presented in ESCA, SKCM (Figure 2B). For DSS, high expression of MATN3 was associated with poor prognosis in ACC, BLCA, CESC, COAD, HNSC, KIRC, LGG, LIHC, MESO, PAAD, STAD, UCEC (Figure 2D). For PFI, high expression of MATN3 was associated with poor prognosis in ACC, BLCA, CESC, COAD, HNSC, KIRC, LGG, LIHC, MESO, PAAD, PCPG, PRAD, STAD, UCEC (Figure 2F).

### Molecular and immunological subtyping

**Figure 3:**
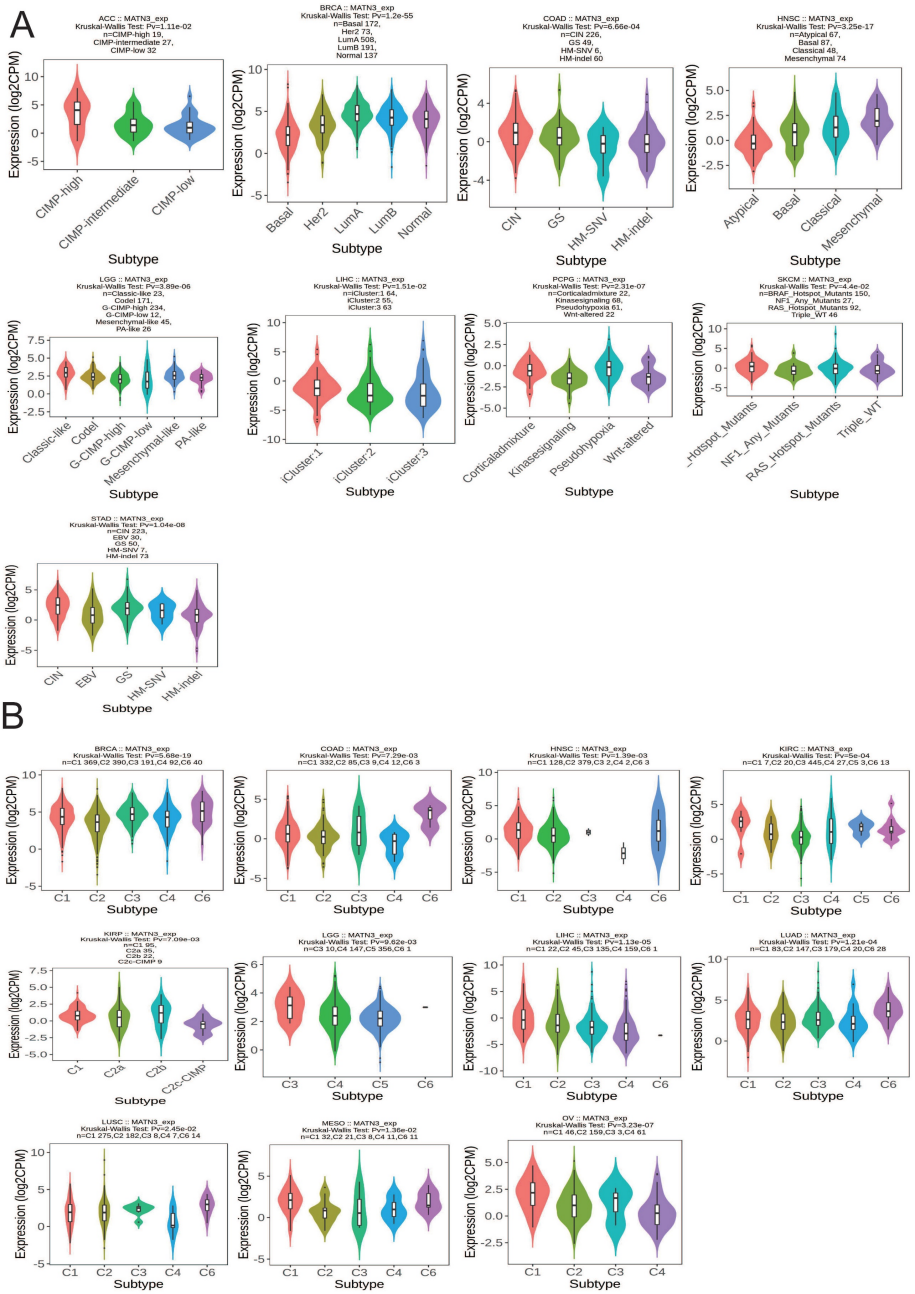
Correlations between MATN3 expression and (**A**) molecular subtypes and (**B**) immune subtypes across TCGA cancer types. CIN, chromosomal instability; GS, genomically stable; POLE, Polymerase ε; EBV, Epstein-Barr virus.C1: wound healing, C2: IFN-gamma dominant, C3: inflammatory, C4: lymphocyte depleted, C5: immunologically quiet, and C6: TGF-b dominant.

We observed that the molecular subtypes of these tumors in ACC, BRCA, COAD, HNSC, LGG, LIHC, PCPG, SKCM, and STAD were correlated with the expression of MATN3.MATN3 expression was higher in CIMP-high in ACC. For BRCA, MATN3 expression was highest in Luma molecular subtype. MATN3 expression was highest in CIN molecular subtype in COAD and Mesenchymal molecular subtype in HNSC. For LGG, MATN3 expression was higher in Classical-like molecular isoforms than in other isoforms. MATN3 expression was higher in the iCluster:1 molecular isoform of LIHC than in other molecular isoforms, in the Pseudohypoxia molecular isoform of PCPG than in other isoforms, and in the Hotspot-Mutans molecular isoform of SKCM than in other isoforms. MATN3 expression was highest in the CIN molecular isoform of COAD and in the Mesenchymal molecular isoform of HNSC. MATN3 expression was upregulated in the CIN molecular subtype of STAD (Figure 3A). The immune subtypes of cancer were closely related to the effect of immunotherapy, and MATN3 expression was significantly correlated with BRCA, COAD, HNSC, KIRC, KIRP, LGG, LIHC, LUAD, LUSC, MESO, and OV (Figure 3B). For BRCA, LUAD, and LUSC, MATN3 was expressed higher in immune subtype C6 than other immune subtypes. In HNSC, KIRC MATN3 expression was higher in immune subtype C1. For KIRP, the expression of MATN3 was higher in C2b than other immune subtypes. The expression of C3 in LGG was higher than other subtypes. For LIHC, MESO, and OV, MATN3 expression was highest in the C1 immunization subtype.

### MATN3 expression and immune checkpoint genes, immunomodulatory genes, and drug sensitivity

**Figure 4:**
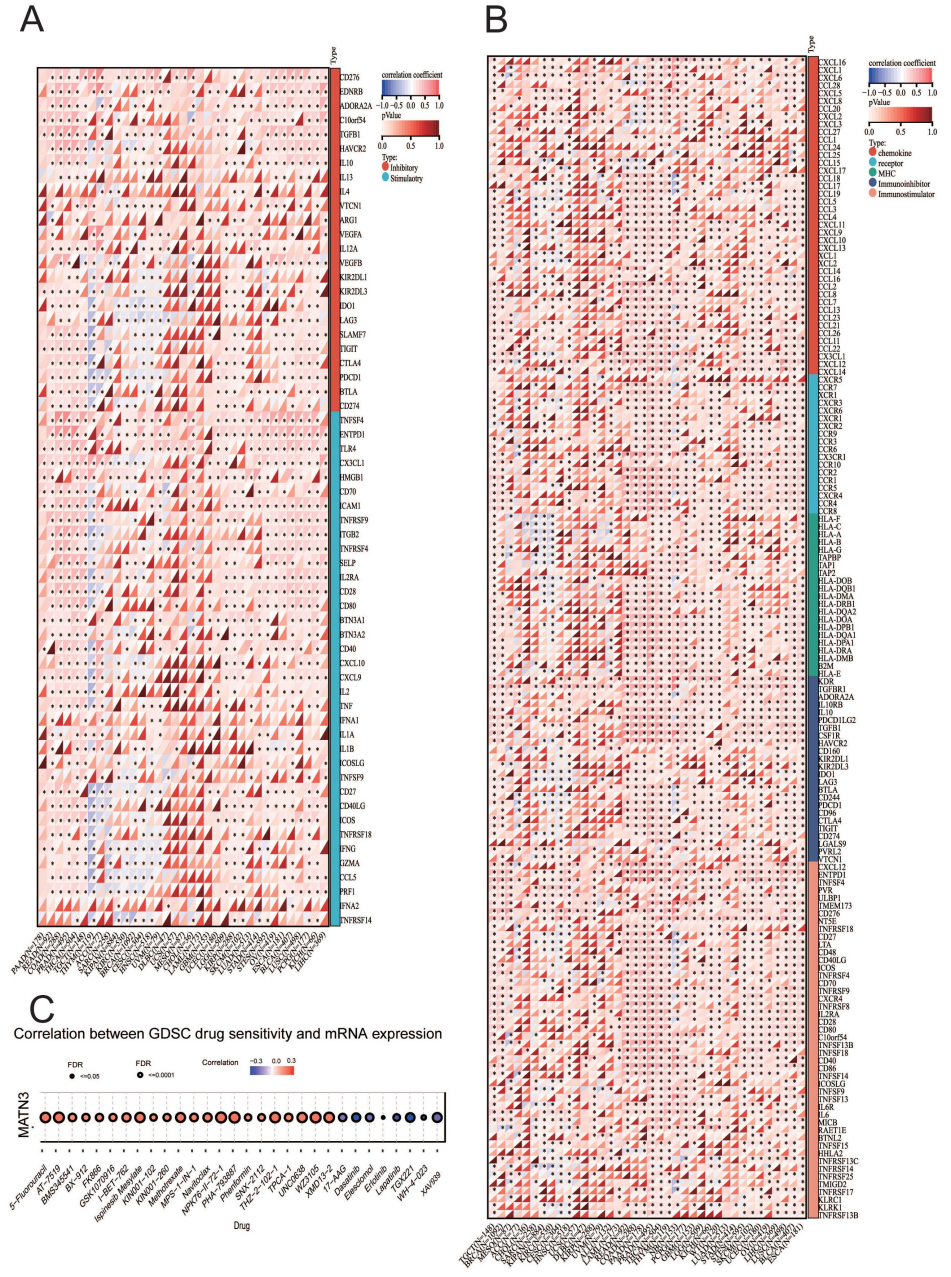
Correlation between MATN3 and immune regulatory genes, immune checkpoints genes, drug sensitivity in pan-cancer. (**A**) Correlation between MATN3 and immune regulatory genes (**B**) Correlation between MATN3 and immune checkpoints (**C**) Association of GSCALite-based expression of MATN3 and related genes with drug sensitivity.

The expression of immune checkpoint genes is closely related to the efficacy of do immunotherapy for cancer, so we explored the correlation between immune checkpoint genes, immunoregulatory genes and MATN3 expression. The results showed that MATN3 expression was positively correlated with cytotoxic T lymphocyte antigen 4 (CTLA4), CD274 (PD-L1), programmed cell death 1 (PDCD1 or PD1), and lymphocyte activation gene 3 (LAG3) in the majority of cancer types, but the opposite was true for THYM, which was negatively correlated with most of the immune checkpoint genes (Figure 4A). We also found that MATN3 was positively correlated with the majority of immunomodulatory genes (Figure 4B). The expression of MATN3 was positively correlated with 5-Fluorouracil, Ispinesb Mesylate, Methotrexate, and Navitoclax, suggesting that high expression of MATN3 may lead to drug resistance. The expression of MATN3 was positively correlated with the expression of Dasatinib, Elesclomol, Erlotinib, and Lapatinib was negatively correlated, suggesting that MATN3 may promote sensitization to this group of drugs (Figure 4C).

### MATN3 and immune infiltration and gene mutations

**Figure 5:**
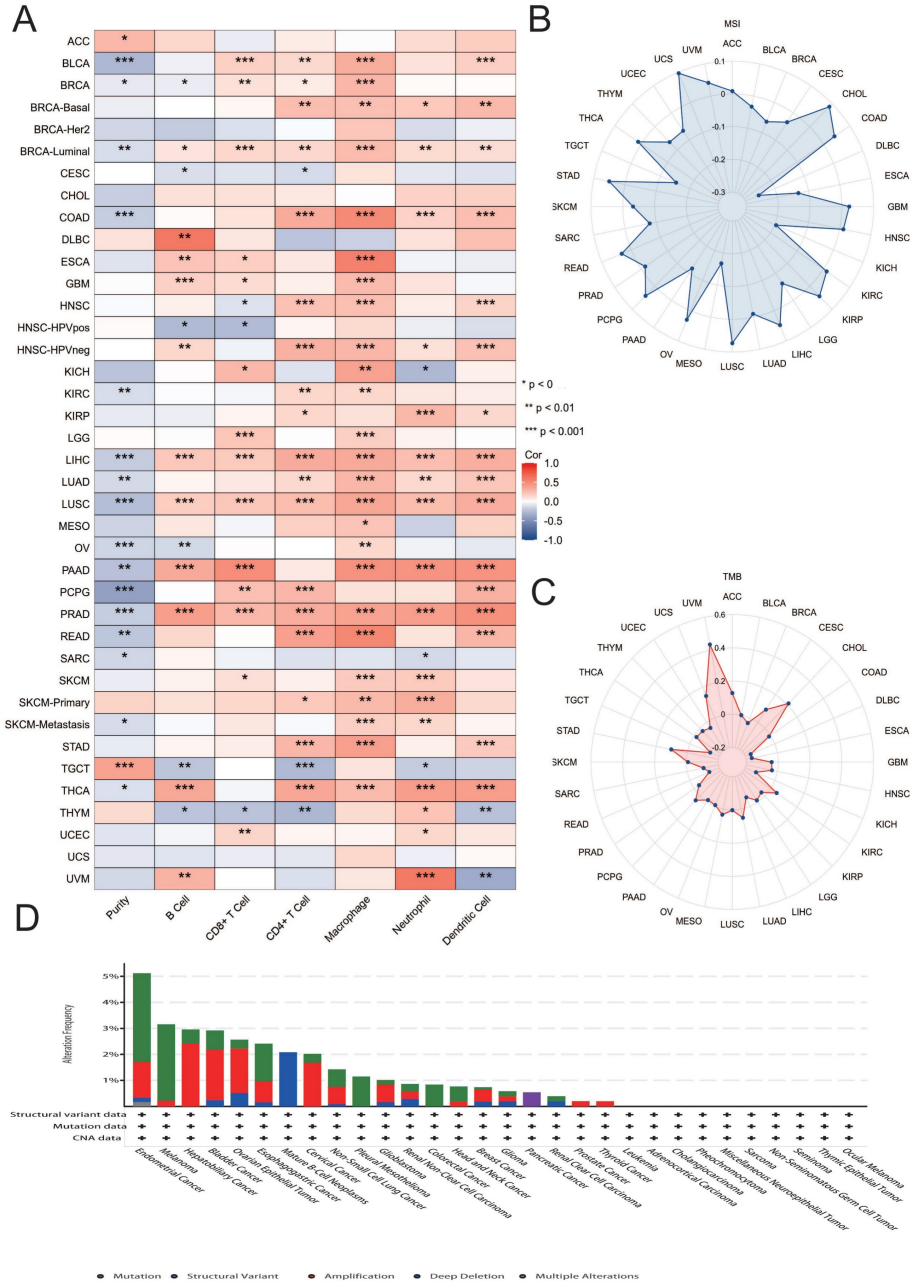
Correlation between MATN3 and immune cells, TMB, MSI, Genetic alterations in pan-cancer. (**A**) Heatmap of correlation between MATN3 expression and 6 tumor-infiltrating cells. (**B**) The correlation of MATN3 expression with TMB. (**C**) The correlation of MATN3 expression with MSI. (**D**) Genetic alterations of MATN3 in pan-cancer using the cBioPortal.

We explored the correlation between MATN3 expression and 6 types of immune-related cells (B cells, CD4+ T cells, CD8+ T cells, neutrophils, macrophages, and dendritic cells) in 33 tumors in the TIMER database, and the results are presented in the form of heatmaps. The results showed that there was a correlation between the expression of MATN3 in BRCA-Luminal, LIHC, LUSC, PRAD and all six immune cells. Among them, the most obvious positive correlation between MATN3 expression and immune cells could be observed in DLBC, and the most obvious negative correlation between MATN3 expression and immune cells was observed in PCPG (Figure 5A). In addition, microsatellite instability (MSI) as well as tumor mutational load (TMB) can be used to predict tumor immunotherapy. we found that the expression of MATN3 in CHOL, COAD, GBM, HNSC, KIRC, KIRP, LIHC, LUSC, OV, PCPG, READ, STAD, THCA, UCS, UVM was positively correlated with MSI, and negatively correlated with BLCA, BRCA, CESC, DLBC, ESCA, KICH, LGG, MESO, PRAD, SARC, SKCM, TGCT, THYM, and UCEC (Figure 5B). The expression of MATN3 was found to be positively correlated in ACC, CESC, CHOL, KIRC, LUAD, MESO, PCPG, SATD, UCS, and TMB of UVM was positively correlated, and negatively correlated with ESCA, DLBC, GBM, HNSC, KICH, KIRP, LIHC, READ, SARC, TGCT, THYM, and UCEC (Figure 5C). We found that the mutation frequency was highest (>6%) in Endometrial cancer, which was dominated by Mutation, followed by Melanoma, whose mutation type was dominated by Mutation, and Hepatobliliary Cancer and Bladder Cancer, whose mutation frequency was similar, both of which were dominated by Amplification mutation type predominated (Figure 5D).

### MATN3 and methylation

**Figure 6:**
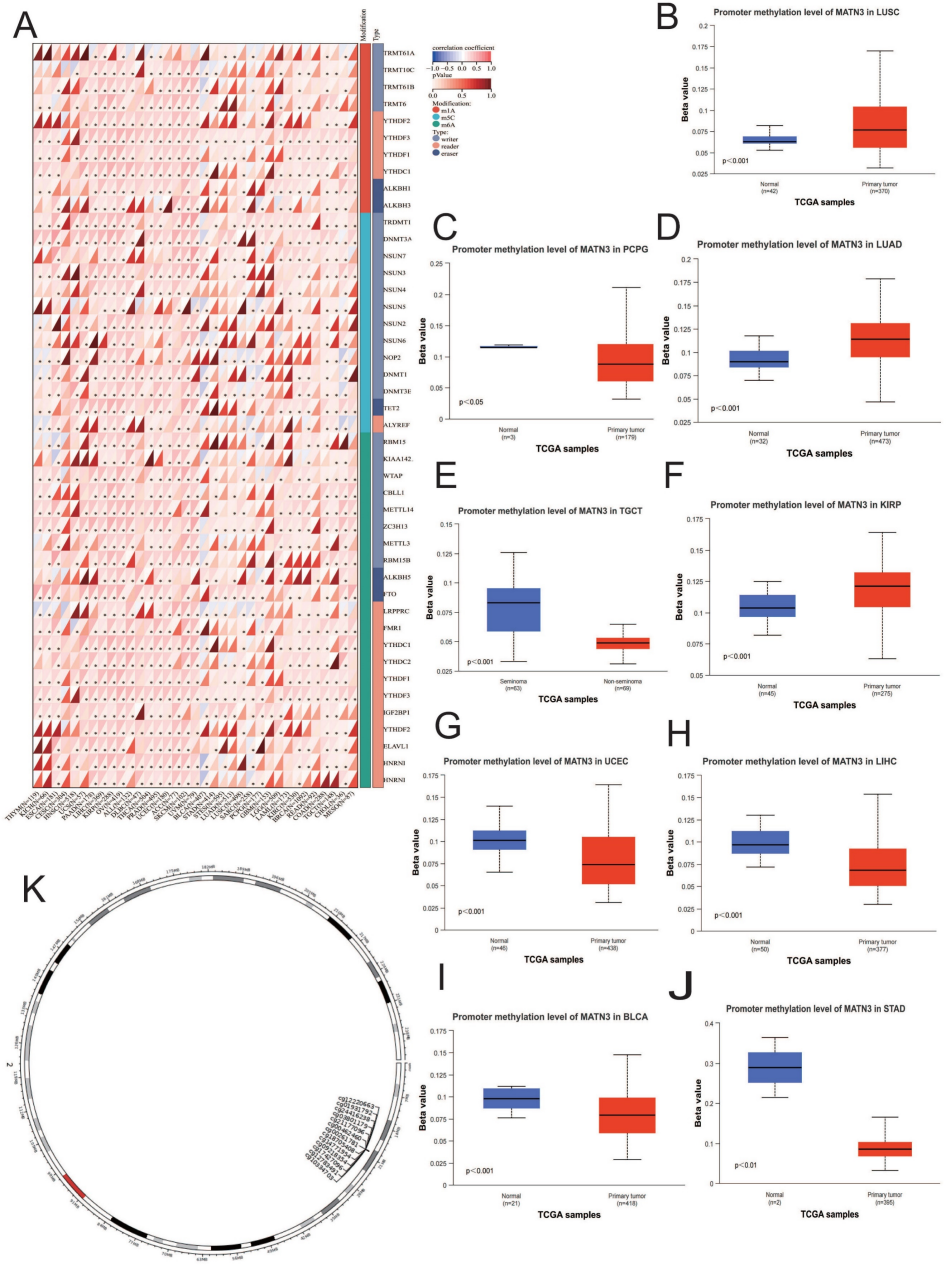
Relationship of MATN3 with methylation and methyltransferase. (**A**) Chromosomal distribution of the methylation probes associated with MATN3.(**B**-**J**) Promoter methylation level of MATN3 in BLCA, KIRP, LIHC, LUAD, LUSC, PCPG, STAD, TGCT and UCEC. (**K**) The correlation between MATN3 expression and m1A, m5C, m6A regulatory genes. *: p < 0.05.

We observed that MATN3 expression at the promoter methylation level was higher in tumor tissues of KIRP (Figure 6F), LUAD (Figure 6D), and LUSC (Figure 6B) than in normal tissues of these cancer types, and was opposite in BLCA (Figure 6I), LIHC (Figure 6H), PCPG (Figure 6C), STAD (Figure 6J), TGCT (Figure 6E), and UCEC (Figure 6G). in which the opposite was true. The differences in the methylation levels of the MATN3 promoter explained, to some extent, the differences in MATN3 expression in some tumors. The correlation analysis of MATN3 expression with methylation modifier genes (m1A, m5C, m6A) showed that (Figure 6A): the OVs were positively correlated with methylation modifier genes in all of them, and the PAAD, LIHC, KIRP, PRAD, UCEC, ACC, and SKCM were positively correlated with the majority of methylation modification genes, and GBM, LAML, and LGG were less correlated with methylation modification genes. In addition, we explored the chromosomal distribution of MATN3-associated methylation probes (Figure 6K), and the 13 methylation probes were: cg21177096, cg00261781, cg00462460, cg01931792, cg03801179, cg09218354, cg10334703, cg12220663, cg12783491, cg14771954, cg17427096, cg18705408, cg21177096, cg24416238. we also explored the relationship between MATN3 methylation and survival (Table 1), which showed that hypermethylated cg21177096 showed worse prognosis in ACC, KIRP, LGG, UCEC, UCS, and good prognosis in COAD, KIRC, LUSC. Hypermethylated cg00261781 shows worse prognosis in KIRP, MESO and good prognosis in GBM, KIRC, LGG, SKCM. Hypermethylated cg00462460 shows worse prognosis in CESC, KIRP and good prognosis in BRCA, HNSC, KIRC, LGG. Hypermethylated cg01931792 shows worse prognosis in BLCA, LIHC and good prognosis in KIRC, KIRP, LGG, MESO, UVM. Hypermethylated cg03801179 shows worse prognosis in KIRP, SKCM and good prognosis in BRCA, HNSC, KIRC.

Hypermethylated cg09218354 shows worse prognosis in KIRP, LIHC, UCEC, UCS and good prognosis in BRCA, COAD, GBM, HNSC, LGG, UVM. Hypermethylated cg10334703 shows worse prognosis in HNSC, LUAD, UCEC, UVM and good prognosis in LGG, SKCM. Hypermethylated cg12220663 shows worse prognosis in COAD, KIRC, LUAD and good prognosis in ACC, KIRP, LGG, UVM. Hypermethylated cg12783491 shows worse prognosis in KIRP, SKCM, UCS and good prognosis in BRCA, PAAD. Hypermethylated cg14771954 showed worse prognosis in UCEC and good prognosis in BRCA, HNSC, LGG. Hypermethylated cg17427096 shows worse prognosis in UCEC, UVM and good prognosis in HNSC, LGG. Hypermethylated cg18705408 shows worse prognosis in CESC, KIRP, UCEC and good prognosis in GBM, HNSC, KIRC, LGG, SKCM. Hypermethylated cg21177096 shows worse prognosis in ACC, KIRP, UCS and good prognosis in COAD, KIRC, LGG, LUSC. Hypermethylated cg24416238 shows worse prognosis in BLCA, HNSC, KIRP and good prognosis in KIRC, LGG, UCS.

### Enrichment analysis

**Figure 7:**
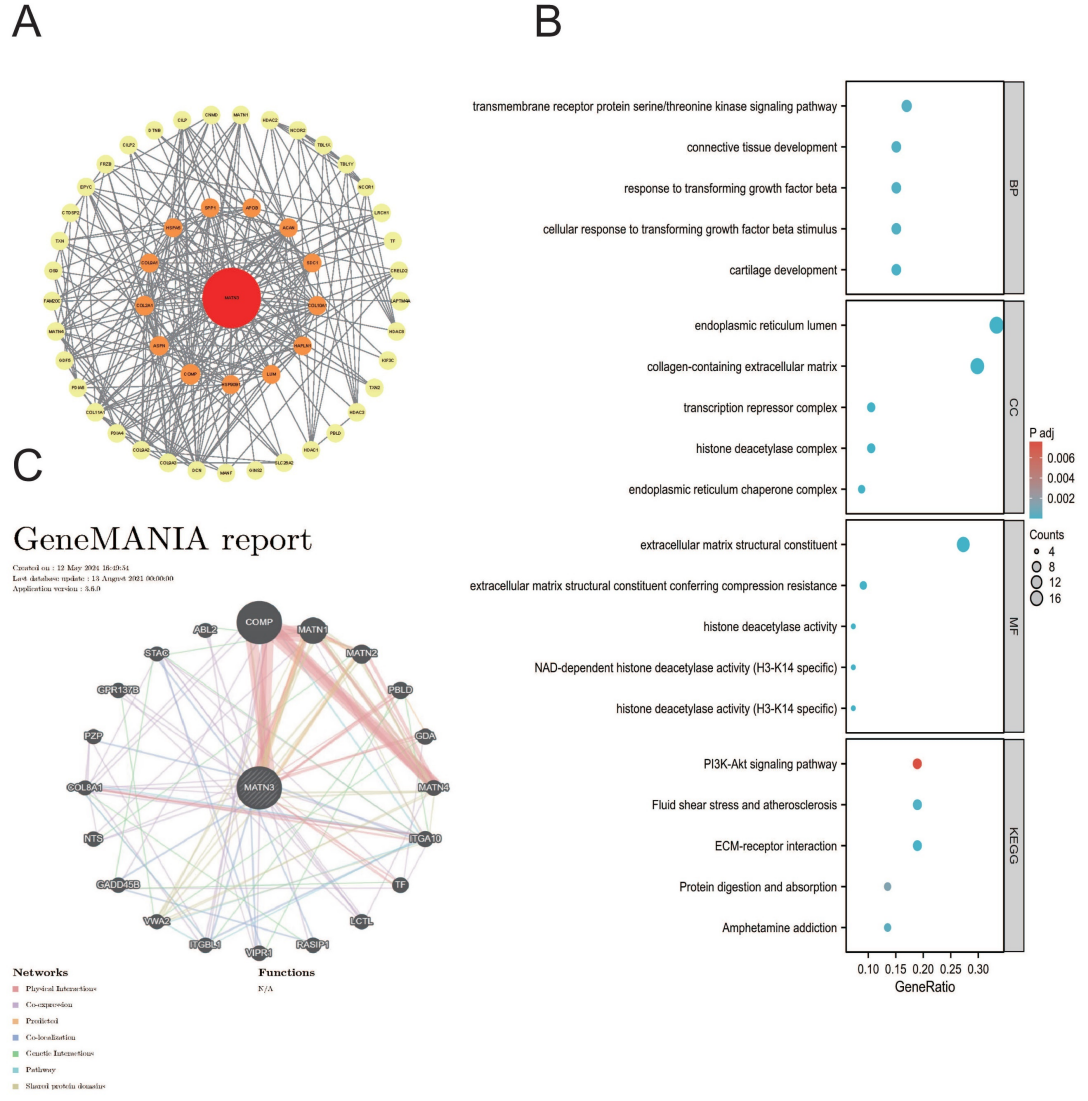
Functional enrichment and co-expression networks of MATN3 at the gene and protein level. (**A**) PPI network. Colors are used to distinguish the relevance of MATN3 to other proteins, with red representing high relevance and green representing low relevance. (**B**) visual network of GO and KEGG analyses. (**C**) GGI network.

We found that MATN3 may be associated with prognosis and immunity in different cancer patients in our previous explorations, and we used the STRING database and the GeneMANIA database to construct the PPI interaction network (Figure 7A) as well as the GGI interaction network (Figure 7C) of MATN3. Based on these related genes we performed Gene Ontology (GO) and Kyoto Encyclopedia of Genes and Genomes (KEGG) enrichment analyses (Figure 7B), and Biological Process (BP) enrichment analyses showed: transmembrane receptor protein serine/threonine kinase signaling pathway, connective tissue development, response to transforming growth factor beta, cellular response to transforming growth factor beta stimulus, cartilage development; molecular function (MF) enrichment analysis showed: endoplasmic reticulum lumen, collagen-containing extracellular matrix, transcription repressor complex, histone deacetylase complex, endoplasmic reticulum chaperone complex; cellular component (CC) enrichment analysis showed: extracellular matrix structural constituent, Extracellular matrix structural constituent, extracellular matrix structural constituent conferring compression resistance, histone deacetylase activity, NAD-dependent histone deacetylase activity (H3-K14 specific), histone deacetylase activity (H3-K14 specific). KEGG pathway analysis revealed that PI3K-Akt signaling pathway, Fluid shear stress and atherosclerosis, ECM-receptor interaction, Protein digestion and absorption, and Amphetamine addiction. we found that MATN3 may be involved in the regulation of tumor development through its effect on the PI3K-Akt signaling pathway. We found that MATN3 may be involved in the regulation of tumor development through its effect on the PI3K-Akt signaling pathway.

### Immunohistochemical analysis

**Figure 8:**
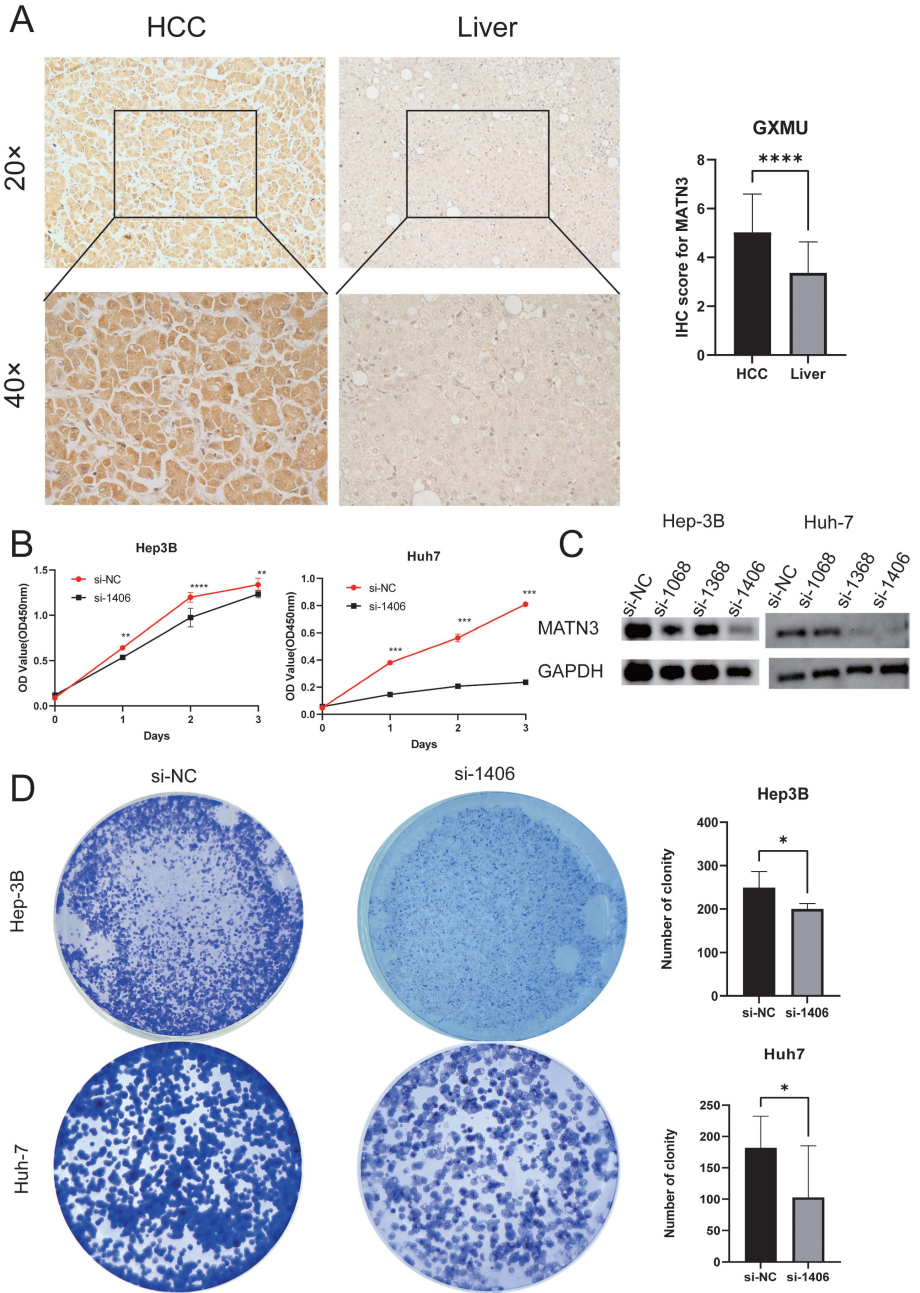
Downregulation of MATN3 can reduce the proliferation ability. (**A**) MATN3 protein expression in LIHC and normal tissues by immunohistochemistry. (**B**) The Cell Counting Kit-8 (CCK-8) assay showed that knockdown of MATN3 inhibited HCC cell proliferation. (**C**) Western blot analysis of MATN3 protein expression levels in the si-1406 and si-NC groups. (**D**) Representative images of colony in the si-NC and si-1406 groups of the indicated cells, with corresponding histogram.

We obtained pathology specimens from 30 patients with hepatocellular carcinoma from the Department of Hepatobiliary Surgery at the First Affiliated Hospital of Guangxi Medical University for immunohistochemical analysis. Semi-quantitative scores were used to evaluate the immunohistochemistry results. The findings indicated that MATN3 protein was significantly overexpressed in hepatocellular carcinoma tissues compared to normal liver tissues (Figure 8A), and the difference in immunohistochemical staining scores between hepatocellular carcinoma tissues and normal liver tissues was statistically significant.

### Downregulation of MATN3 can reduce the proliferation and migration ability of liver cancer cells

**Figure 9:**
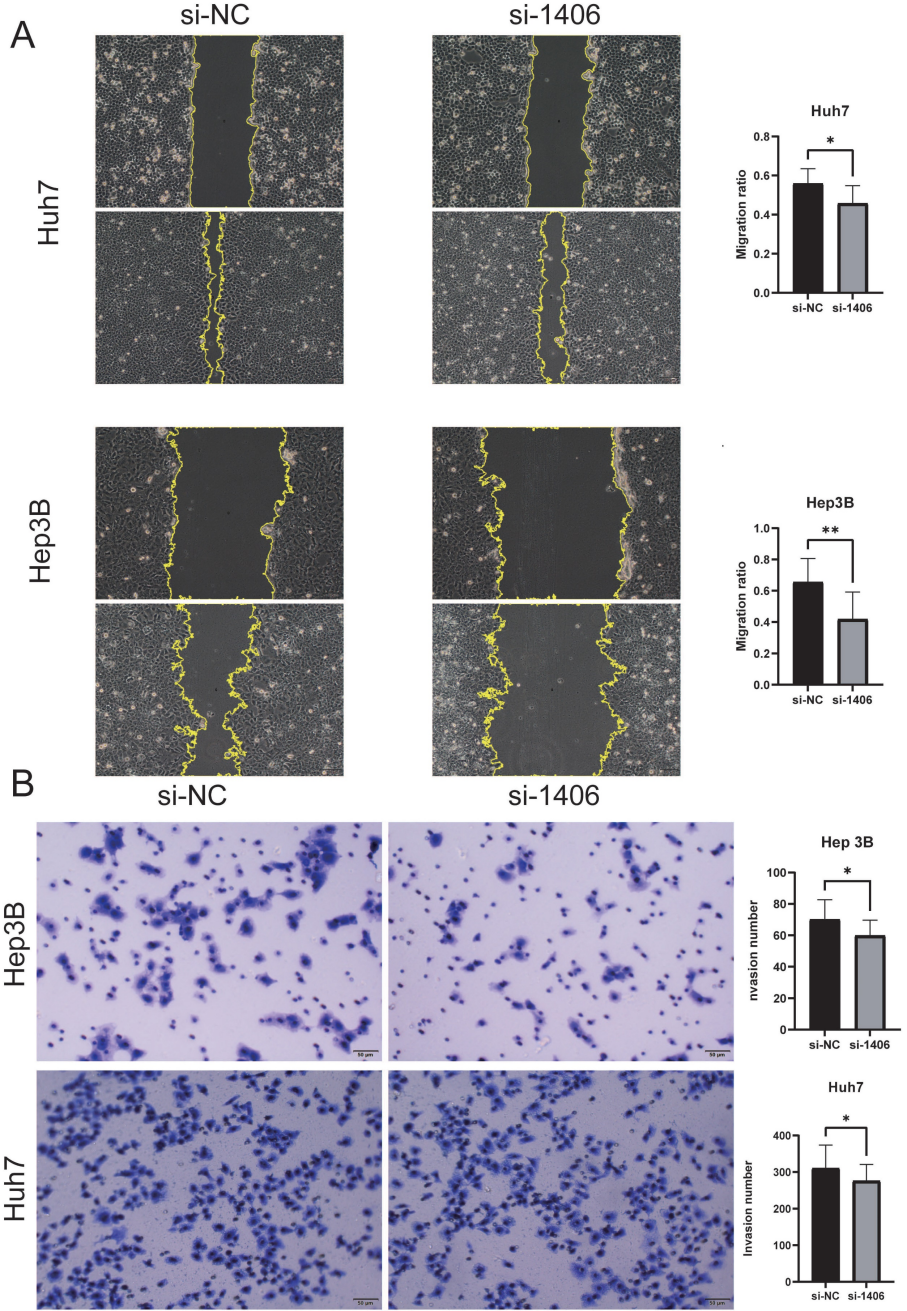
Downregulation of MATN3 can reduce the migration ability. (**A**) Wound healing assay revealing that MATN3 overexpression promotes Hep3B and Huh7 cell migration. Data are presented as the mean ± SD of three independent experiments, ***P < 0.001, Student's t-test. (**B**) Transwell migration assays demonstrating that knockdown of MATN3 decreases the migratory abilities of HCC cells. The migrated cells were counted. Data are presented as the mean ± SD of three independent experiments, ***P < 0.001, Student's t-test.

The knockdown efficiency was evaluated by western blotting (Figure 8C), and the proliferation assay CCK-8 (Figure 8B) and colony formation assay showed that the proliferation ability of si-MATN3 group was reduced compared with that of NC group (Figure 8D), and the Cell migration assay (Figure 9A) and wound healing assay (Figure 9B) showed that the migratory ability of si-MATN3 group was reduced compared with that of NC group.

## Discussion

Currently, the rapid progress of multi-omics analysis technology enables people to understand the occurrence and development of cancer from more angles. mRNA, non-coding RNA, protein and other biomarkers^24^ may be present in cancer tissues, normal tissues, blood, urine and other parts of the patient's body, and their stable and detectable nature is widely used in early diagnosis and prognosis of cancer patients^25^. MATN3 is a member of the matrilin protein family, which has received extensive attention in the field of bone and cartilage. MATN3 is a member of the matrilin family of proteins, which has received much attention in the skeletal as well as cartilage fields, where mutations may affect skeletal dysplasia^26^ and may be a risk factor for arthritis of the hand^27^. MATN3 is still underexplored in the field of cancer, and it has been reported that MATN3 plays an important role in the development of gastric adenocarcinomas^28^. As a classical secretory protein, MATN3 can hinder immune cell infiltration and promote tumor progression, but there is still a lack of relationship between MATN3 expression and immune infiltration at pan-cancer level, so we have studied the relationship between MATN3 expression and immune infiltration, which is consistent with the previous conclusion. It is helpful to further understand the mechanism of immune infiltration and MATN3 expression in different tumors. Based on TCGA and GTEx databases, our work found that MATN3 was significantly up-regulated in 15 cancer types, and MATN3 expression was relatively high in a variety of cancer types in relatively high T stage, relatively high N stage, relatively high M stage, and higher histologic grading. We performed immunohistochemistry on hepatocellular carcinoma as a means of confirming MATN3 expression in tumor tissue and normal tissue samples. We found that MATN3 protein expression was significantly upregulated in tumor tissues of hepatocellular carcinoma compared to normal paracancerous tissues^29^. Survival analysis revealed that high expression of MATN3 was worse in OS, DSS, and PFI in ACC, BLCA, CESC, HNSC, KIRC, LGG, LIHC, MESO, PCPG, PRAD, STAD, and UCEC. High expression of MATN3 predicted poor prognosis in most cancer types. Our work also observed a close association of MATN3 expression in 9 different molecular subtypes^30^ and 11 different immune subtypes^31^, which may be an entry point for subsequent studies. In addition, we explored the correlation of MATN3 expression with immune checkpoint genes and immunomodulatory genes as well as the relationship between MATN3 expression and drug sensitivity. Immune checkpoint genes are important targets of immune checkpoint inhibitors for cancer treatment^32^, and we found that MATN3 expression was significantly correlated with immune checkpoint genes (CTLA4, PD-L1, PD1). The expression of MATN3 was negatively correlated with Dasatinib^32^, Elesclomol^33^, Erlotinib^34^, and Lapatinib^35^, suggesting that MATN3 may promote the sensitivity of this part of drugs and play a positive role in cancer treatment.

We also explored the relationship between MATN3 and the infiltration of six types of immune cells, and we found that four types of cancer types, BRCA-Luminal, LIHC, LUSC, and PRAD, were significantly correlated with all six types of immune cells. Microsatellite instability (MSI)^35^ as well as tumor mutational load (TMB)^36^ are instructive for immunotherapy of cancer, and our work found that MSI and TMB are closely associated with most cancer types. We also explored the relationship between gene mutations and cancer, and the results showed that Amplification was the main mutation type^37^. In addition, methylation modification, as one of the most common epigenetic modifications, is also an important step in regulating cancer development^38^, we explored the expression of MATN3 promoter methylation in different cancer types as well as the correlation between MATN3 expression and methylation-associated genes, and also explored the relationship between methylated water and prognosis, and the results showed that MATN3 promoter methylation may play a different roles, and MATN3 expression was positively correlated with most RNA methylation modification genes, which suggests that we MATN3 may be involved in methylation modification and thus in the regulation of tumor development^39^. Enrichment analysis showed that MATN3 was likely to regulate the occurrence and progression of tumors by participating in the PI3K-Akt signaling pathway. This may provide some entry points for subsequent studies to explore the regulatory pathways of MATN3 in cancer. *In vitro* experiments in hepatocellular carcinoma have verified that MATN3 knockdown can inhibit the proliferation and migration of hepatocellular carcinoma, but the specific regulatory mechanism of MATN3 in hepatocellular carcinoma remains to be further studied. In conclusion, this study analyzed the differential expression, diagnostic value, prognostic value, methylation, immune infiltration and enrichment pathway of MATN3 in various cancer types using multiple databases, and MATN3 is expected to be a prognostic and immune biomarker for pan-cancer.

## Conclusion

Our work systematically explored the role of MATN3 in cancer in terms of MATN3 expression and clinical features, diagnostic value, prognosis, gene mutation, drug sensitivity, methylation and its prognosis, immune infiltration, immune checkpoints, TMB, and MSI, and the results showed that MATN3 was significantly correlated with these factors, which could provide a better tool for us to follow up and explore the potential value of MATN3 in cancer. In this study, multiple databases were used to analyze the differential expression, diagnostic value, prognostic value, methylation, immune infiltration and enrichment pathway of MATN3 in a variety of cancer types. Based on previous studies, our study explored the influence of MATN3 on cancer from a broader perspective. Knocking down MATN3 in liver cancer cell lines verified that MATN3 can reduce the proliferation and migration ability of liver cancer cells, which provided a certain basis for further exploration of the effects of MATN3 on cancer.

## Supplementary Material

Supplementary figure and tables.

## Figures and Tables

**Table 1:** Relationship between MATN3 methylated CpG and survival.

	GCP	HR	P value		
ACC	cg21177096	5.268(1.251;22.18)	0.004003342		
	cg12220663	0.445(0.207;0.958)	0.033050964		
	cg01931792	2.321(1.068;5.043)	0.043290041		
BLCA	cg01931792	1.517(1.045;2.204)	0.022453353		
	cg24416238	1.501(1.019;2.211)	0.031771102		
BRCA	cg12783491	0.563(0.36;0.881)	0.008670634		
	cg00462460	0.593(0.396;0.888)	0.009793759		
	cg14771954	0.543(0.33;0.894)	0.010850188		
	cg03801179	0.585(0.338;1.012)	0.041261465		
	cg09218354	0.66(0.44;0.991)	0.041569449		
CESC	cg00462460	1.893(1.018;3.52)	0.030931498		
	cg18705408	1.603(1;2.57)	0.04764151		
COAD	cg12220663	1.848(1.112;3.072)	0.01457385		
	cg21177096	0.571(0.347;0.938)	0.023638182		
	cg09218354	0.592(0.362;0.969)	0.042481875		
GBM	cg00261781	0.53(0.349;0.806)	0.002782668		
	cg18705408	0.53(0.337;0.834)	0.007945736		
	cg09218354	0.575(0.365;0.906)	0.02133314		
HNSC	cg03801179	0.545(0.384;0.773)	0.000294804		
	cg18705408	0.597(0.423;0.84)	0.001854571		
	cg10334703	1.683(1.19;2.38)	0.001887737		
	cg24416238	1.498(1.079;2.08)	0.012185988		
	cg17427096	0.698(0.519;0.938)	0.01454275		
	cg14771954	0.702(0.511;0.963)	0.02396785		
	cg09218354	0.711(0.516;0.979)	0.031364099		
	cg00462460	0.719(0.518;0.998)	0.041659608		
KIRC	cg12220663	2.544(1.468;4.407)	0.000221572		
	cg00462460	0.475(0.299;0.754)	0.000802375		
	cg18705408	0.531(0.339;0.83)	0.003596935		
	cg21177096	0.575(0.389;0.851)	0.005135328		
	cg03801179	0.516(0.306;0.868)	0.007512144		
	cg01931792	0.544(0.327;0.904)	0.012244874		
	cg24416238	0.629(0.389;1.018)	0.048748302		
	cg00261781	0.678(0.459;1.002)	0.049702631		
KIRP	cg12783491	2.669(1.433;4.969)	0.002587075		
	cg21177096	2.691(1.368;5.295)	0.002621146		
	cg01931792	0.379(0.204;0.706)	0.00289106		
	cg00462460	2.546(1.351;4.796)	0.005755506		
	cg24416238	2.337(1.238;4.413)	0.007978722		
	cg18705408	2.376(1.254;4.502)	0.01074089		
	cg00261781	2.236(1.177;4.247)	0.018586969		
	cg10334703	0.455(0.24;0.863)	0.019813683		
	cg12220663	0.309(0.095;1.007)	0.021727231		
	cg09218354	2.162(1.122;4.164)	0.027707707		
	cg03801179	1.97(1.035;3.747)	0.035250812		

## Abbreviations

ACC: adrenocortical carcinoma
BLCA: bladder urothelial carcinoma
BRCA: breast invasive carcinoma
CESC: cervical squamous cell carcinoma and endocervical adenocarcinoma
CHOL: cholangiocarcinoma
COAD: colon adenocarcinoma
DLBC: lymphoid neoplasm diffuse Large B-cell lymphoma
ESCA: esophageal carcinoma
GBM: glioblastoma multiforme
HNSC: head and neck squamous cell carcinoma
KICH: kidney chromophobe
KIRC: kidney renal clear cell carcinoma
KIRP: kidney renal papillary cell carcinoma
LAML: acute myeloid leukemia
LGG: brain lower grade glioma
LIHC: liver hepatocellular carcinoma
LUAD: lung adenocarcinoma
LUSC: lung squamous cell carcinoma
MESO: mesothelioma
OV: ovarian serous cystadenocarcinoma
PAAD: pancreatic adenocarcinoma
PCPG: pheochromocytoma and paraganglioma
PRAD: prostate adenocarcinoma
READ: rectum adenocarcinoma
SARC: sarcoma
SKCM: skin cutaneous melanoma
STAD: stomach adenocarcinoma
TGCT: testicular germ cell tumors
THCA: thyroid carcinoma
THYM: thymoma
UCEC: uterine corpus endometrial carcinoma
UCS: uterine carcinosarcoma
UVM: uveal melanoma
