# Supplementary material for: The Role of MATN3 in Cancer Prognosis and Immune Infiltration Across Multiple Tumor Types
Source: J Cancer. 2025 Jan 27;16(5):1519–37. doi: 10.7150/jca.103523 (PMC11843250; doi:10.7150/jca.103523)
Supplement: Supplementary file 1 — Supplementary figure and tables. [file jcav16p1519s1.pdf]

Figure legends

FigureS1

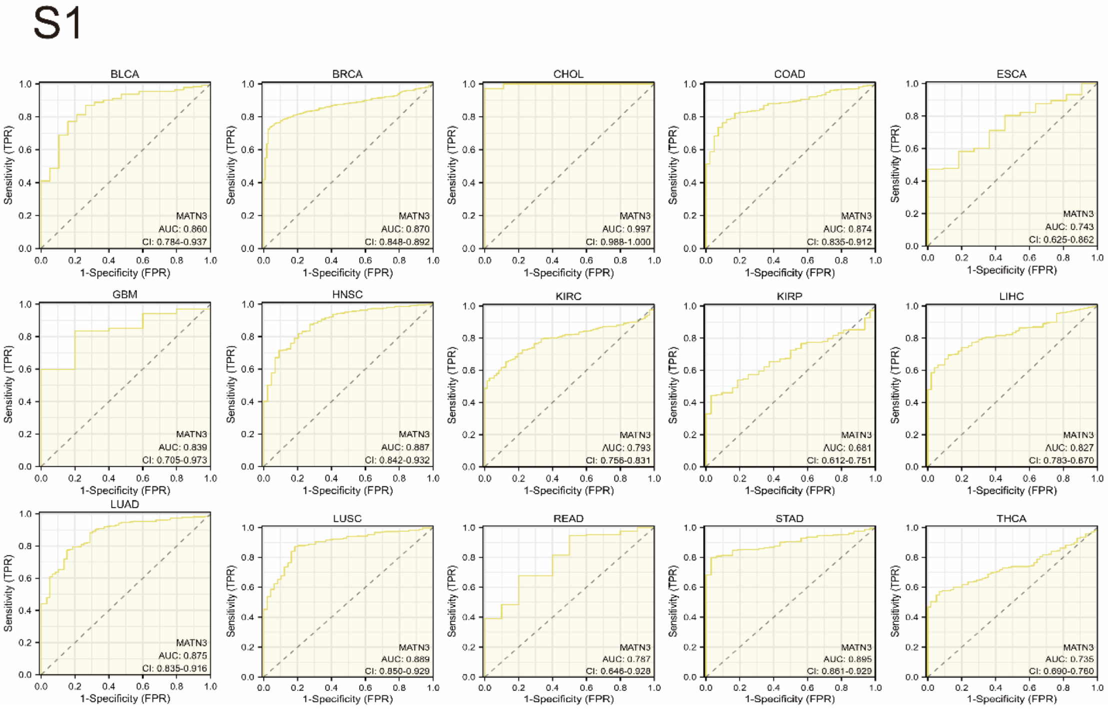

Figure legends

FigureS1. Diagnostic value of MATN3 in 15 cancers types.

Tables

Table S1

The clinical parameters of 30 patients

Patients	Gender	Age	Cancer type	CNLC	BCLC	Child pugh	AFP(ng/ml)	Vascular Tumor thrombus	MVI	Smoking history	Alcohol history	HBV	hypertension	diabetes	Heart disease	History of surgery	Other Diseases
1	male	54	HCC	IA	A	A	40.12	0	M0	1	1	1	0	0	0	0	0
2	male	66	HCC	IA	A	A	1560.3	0	M0	0	1	1	0	0	0	1	0
3	female	69	HCC	IB	B	A	152.57	1	M0	0	0	0	0	0	0	0	0
4	female	72	HCC	IIA	B	A	34.14	0	M0	1	1	1	0	0	0	0	0
5	male	51	HCC	IA	A	A	156.34	1	M0	1	1	1	0	0	0	1	0
6	female	70	HCC	IIA	B	A	5.51	0	M0	0	0	1	0	0	0	0	0
7	male	44	HCC	IB	B	A	1.51	0	M1	0	1	1	0	0	0	1	0
8	male	48	HCC	IA	A	A	889.67	1	M0	1	1	0	0	0	0	0	0
9	male	59	HCC	IA	A	A	54.62	0	M0	1	1	1	0	0	0	0	0
10	male	71	HCC	IIB	B	A	3.15	0	M0	0	0	1	1	1	0	0	1
11	male	39	HCC	IA	A	A	46.56	0	M0	0	0	1	0	0	0	0	0
12	male	43	HCC	IA	A	A	2.63	0	M1	1	1	1	0	0	0	0	0
13	male	57	HCC	IA	A	A	78.36	0	M0	1	1	1	0	0	0	1	0
14	female	72	HCC	IIB	B	A	123.36	0	M0	1	1	1	0	0	0	0	0
15	male	35	HCC	IA	A	A	65.39	0	M0	1	0	1	0	0	0	0	0
16	female	48	HCC	IIB	B	A	477.32	0	M0	0	0	1	0	0	0	1	0
17	male	34	HCC	IA	A	A	68.36	0	M0	1	1	1	0	0	0	0	0
18	male	57	HCC	IA	A	A	135.36	0	M0	1	1	1	0	0	0	0	0
19	female	72	HCC	IA	A	A	89.57	0	M0	0	0	1	0	0	0	0	0
20	male	53	HCC	IA	A	A	75.5	1	M0	0	0	1	0	0	0	0	0
21	female	44	HCC	IA	A	A	2.89	0	M0	0	0	1	0	0	0	0	0
22	male	45	HCC	IIB	B	A	89.45	0	M1	0	1	1	0	0	0	1	0
23	male	60	HCC	IA	A	A	45.68	0	M0	1	1	0	1	0	0	1	0
24	female	59	HCC	IA	A	A	84.54	0	M0	0	0	1	0	0	0	0	0
25	female	67	HCC	IIB	B	A	356.45	0	M0	0	0	1	0	0	0	0	0
36	male	44	HCC	IA	A	A	56.54	0	M0	1	1	1	0	0	0	0	0
37	female	69	HCC	IA	A	A	86.65	0	M1	0	0	1	0	0	0	0	0
38	female	41	HCC	IA	A	A	68.98	0	M0	0	0	1	0	0	0	0	0
39	male	56	HCC	IIB	B	A	59.67	0	M0	1	1	1	0	0	0	0	0
30	male	67	HCC	IA	A	A	167.65	0	M0	1	1	1	0	0	0	0	0

Table S2

Logistic regression analysis of MATN3 expression.

Characteristics	Total (N)	OR (95% CI)	P value
Age (> 60 vs. <= 60)	373	0.602 (0.400 – 0.907)	**0.015**
Gender (Male vs. Female)	374	0.843 (0.546 – 1.300)	0.439
Race (White vs. Asian&Black or African American)	362	0.976 (0.646 – 1.474)	0.909
BMI (> 25 vs. <= 25)	337	0.920 (0.600 – 1.410)	0.701
AFP(ng/ml) (> 400 vs. <= 400)	280	1.446 (0.829 – 2.524)	0.194
Child-Pugh grade (B&C vs. A)	241	0.992 (0.411 – 2.392)	0.985
Pathologic T stage (T3&T4 vs. T1&T2)	371	1.723 (1.069 – 2.777)	**0.025**
Pathologic N stage (N1 vs. N0)	258	71472866.1094 (0.000 – Inf)	0.995
Pathologic M stage (M1 vs. M0)	272	2.912 (0.299 – 28.345)	0.357
Pathologic stage (Stage III&Stage IV vs. Stage I&Stage II)	350	1.833 (1.126 – 2.985)	**0.015**
Histologic grade (G3&G4 vs. G1&G2)	369	1.312 (0.858 – 2.005)	0.21
Tumor status (With tumor vs. Tumor free)	355	0.918 (0.603 – 1.398)	0.691
Vascular invasion (No vs. Yes)	318	0.813 (0.512 – 1.291)	0.379

Table S3

Sequence of interfering RNA for MATN3.

Oligo name		Sequence (5’-3’)
MATN3-1368-s	sense strand	GCUUGUGGAUGUGAAGCUA
	antisense strand	UAGCUUCACAUCCACAAGC
MATN3-1068-s	sense strand	ACUUGUUCAGCUCAAGAUA
	antisense strand	UAUCUUGAGCUGAACAAGU
MATN3-1406-s	sense strand	GGUCAGCUCGUAUCUUCAA /
	antisense strand	UUGAAGAUACGAGCUGACC
NC	sense strand	UUCUCCGAACGUGUCACGUTT
	antisense strand	ACGUGACACGUUCGGAGAATT
FAM-NC	sense strand	UUCUCCGAACGUGUCACGUTT
	antisense strand	ACGUGACACGUUCGGAGAATT
